# The association of breast cancer patients survival and prior menopausal hormone therapy in women with type 2 diabetes

**DOI:** 10.1038/s41598-024-65916-2

**Published:** 2024-07-16

**Authors:** Mayu Hosio, Elina Urpilainen, Ari Hautakoski, Martti Arffman, Reijo Sund, Anne Ahtikoski, Ulla Puistola, Arja Jukkola, Esa Läärä, Peeter Karihtala

**Affiliations:** 1 Department of Oncology and Radiotherapy, Oulu University Hospital, Wellbeing Services County of North Ostrobothnia, University of Oulu, Oulu, Finland; 2 Cancer and Translational Research Unit, University of Oulu, Oulu, Finland; 3 Medical Research Center, Oulu University Hospital, Wellbeing Services County of North Ostrobothnia, University of Oulu, Oulu, Finland; 4 Department of Obstetrics and Gynecology, Oulu University Hospital, Wellbeing Services County of North Ostrobothnia, University of Oulu, Oulu, Finland; 5 Research Unit of Clinical Medicine, University of Oulu, Oulu, Finland; 6 Department of Public Health and Welfare, Finnish Institute for Health and Welfare, Helsinki, Finland; 7 Institute of Clinical Medicine, University of Eastern Finland, Kuopio, Finland; 8 Fimlab Laboratories, Department of Pathology, University of Tampere, Tampere, Finland; 9 Department of Oncology and Radiotherapy, Tays Cancer Center, Tampere University Hospital, Faculty of Medicine and Health Technology, Tampere University, Tampere, Finland; 10 Research Unit of Mathematical Sciences, University of Oulu, Oulu, Finland; 11 Department of Oncology, Helsinki University Hospital Comprehensive Cancer Center, University of Helsinki, P.O. Box 180, 00029 Helsinki, Finland

**Keywords:** Breast cancer, Cancer epidemiology

## Abstract

We investigated the association of prediagnostic use of menopausal hormone therapy (MHT) with breast cancer survival among women with type 2 diabetes (T2D). The study cohort was identified from a Finnish nationwide diabetes database, and consisted of women with T2D, who were diagnosed with breast cancer between 2000 and 2011 (n = 3189). The patients were classified according to their previous MHT use: systemic MHT, local MHT, and no history of any MHT. The cumulative mortality from breast cancer, cardiovascular diseases, and other causes in three MHT groups was described by the Aalen-Johansen estimator. The cause-specific mortality rates were analyzed by Cox models, and adjusted hazard ratios (HRs) were estimated for the use of MHT. The breast cancer mortality appeared to be lower among systemic MHT users (HR 0.49, 95% Cl 0.36–0.67) compared with non-users of MHT. The mortality from cardiovascular diseases and from other causes of death was found to be lower among systemic MHT users, (HR 0.49, 95% Cl 0.32–0.74), and (HR 0.51, 95% Cl 0.35–0.76), respectively. In conclusion, prediagnostic systemic MHT use is associated with reduced breast cancer, cardiovascular, and other causes of mortality in women with T2D.

## Introduction

Several studies have suggested that breast cancer patients with type 2 diabetes (T2D) have a higher breast cancer-specific and overall mortality rate compared with patients without T2D^1,2^. One reason for this can be that breast cancer patients with T2D may be less likely to receive optimal breast cancer treatments due to T2D-related comorbidities and their complications^3^.

Menopause is associated with a marked decrease in estrogen production, leading to diminished serum estradiol concentrations and hot flashes. Menopausal hormone therapy decreases substantially the frequency and severity of hot flashes^4,5^. The benefit-risk ratio for systemic MHT is generally considered favorable when initiated before 60 years of age or within 10 years of menopause onset, while older women receiving MHT have greater absolute risks of stroke, coronary heart disease, dementia, and venous thromboembolism^6^. Previous breast cancer is a contraindication for systemic MHT. Local MHT is used for vulvovaginal and genitourinary symptoms. Although not as potent as systemic MHT, it improves the quality of life in a substantial proportion of patients and may also be used also in patients with breast cancer history with certain limitations^7,8^.While increased breast cancer risk with menopausal hormone therapy (MHT) is well known^9^, there is some evidence of a reduction in overall mortality among MHT-users^10^. The association between prediagnostic MHT use and cancer-specific and all-cause mortality among breast cancer patients has been found to be favorable compared to non-MHT users in most cohort studies, although results have been variable^11–18^. Randomized controlled trials have also reported that MHT use reduces the incidence of T2D in women^19^. Additionally, MHT use has been reported to reduce insulin resistance in T2D patients^19^. It has been hypothesized that metabolic changes due to estrogen depletion after menopause may contribute to increased cardiovascular disease (CVD) risk in post-menopausal women^20^. Furthermore, evidence suggests that MHT contributes to decreased CVD risk based on the cardioprotective hypothesis concerning estrogen^21,22^. However, the observational study results regarding an association between MHT and CVD risk vary^21,23,24^.

In the present nationwide register-based cohort study, we investigated the survival of breast cancer patients in relation to the use of prediagnostic MHT in women with T2D. To the best of our knowledge, no previous reports on this special population exist in the literature and most of the previous studies have also combined local and systemic MHT.

## Results

**Figure 1:**
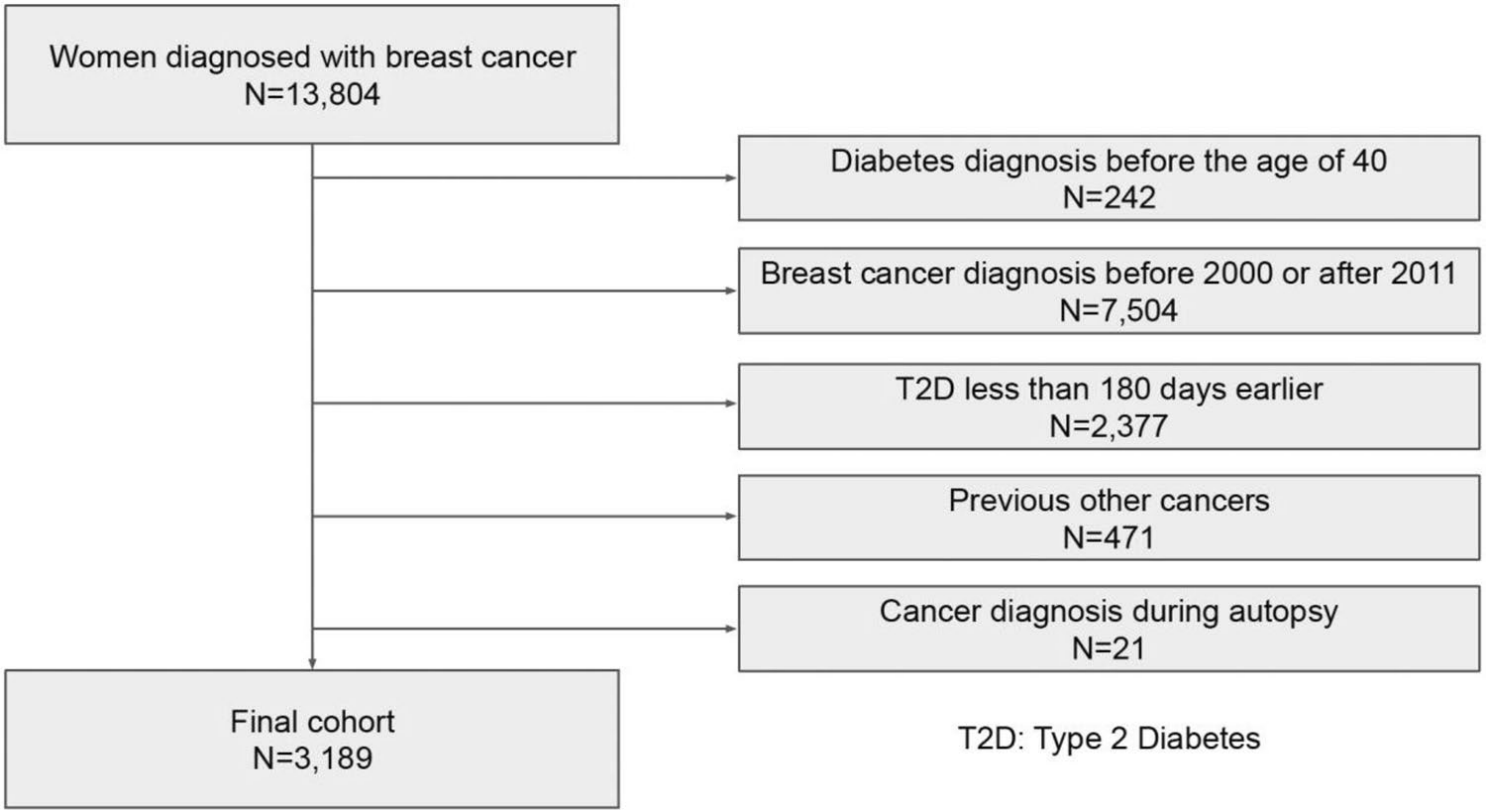
Flowchart of the study.

The final cohort included 3,189 eligible women (aged 41–100 years at the time of breast cancer diagnosis) with breast cancer and T2D (Fig. 1). The median follow-up period was 4.5 years (interquartile range: 2.6–7.3 years). In total, 579 (18%) were classified as systemic MHT users, 310 (10%) as local MHT users, and 2,300 (72%) had no MHT history (Table 1). Systemic MHT users, on average, were younger than women in the local MHT group (Table 1). In total, 1,274 patients died during the follow-up period.

**Table 1:** Distribution of baseline characteristics and outcome status in the different types of menopausal hormone therapy (MHT) groups.

		Menopausal hormone therapy (MHT)			
		None (%)	Systemic (%)	Local (%)	Total (%)
Total	n	2300	579	310	3189
	2000–2003	615 (27)	136 (23)	44 (14)	795 (25)
	2004–2007	706 (31)	179 (31)	100 (32)	985 (31)
	2008–2011	979 (43)	264 (46)	166 (54)	1409 (44)
Age at BC diagnosis	Median	75	65	72	72
(years)	IQR	65–81	60–70	65–79	64–80
Age at BC diagnosis categorised					
(years)	40–59	324 (14)	129 (22)	20 (6)	473 (15)
	60–64	257 (11)	166 (29)	57 (18)	480 (15)
	65–69	287 (12)	138 (24)	58 (19)	483 (15)
	70–74	314 (14)	77 (13)	60 (19)	451 (14)
	75–79	448 (19)	44 (8)	52 (17)	544 (17)
	80–84	368 (16)	17 (3)	38 (12)	423 (13)
	85–100	302 (13)	8 (1)	25 (8)	335 (11)
Duration of diabetes					
	Median	6.8	5.8	7.1	6.6
	IQR	3.2–11.6	2.8–9.9	3.6–12.4	3.1–11.3
Duration of diabetes categorised					
(years)	0.5–< 3	544 (24)	159 (27)	67 (22)	770 (24)
	3–< 6	495 (22)	134 (23)	59 (19)	688 (22)
	6–< 12	716 (31)	196 (34)	105 (34)	1,017 (32)
	12–< 42	545 (24)	90 (16)	79 (25)	714 (22)
Stage					
	Local	1080 (47)	325 (56)	172 (55)	1577 (49)
	Advanced	1048 (46)	226 (39)	119 (38)	1393 (44)
	Unknown	172 (7)	28 (5)	19 (6)	219 (7)

*BC* Breast Cancer, *MHT* Menopausal hormone therapy, *IQR* Interquartile range.

The entries are numbers (percentages in parentheses) unless otherwise stated.

**Figure 2:**
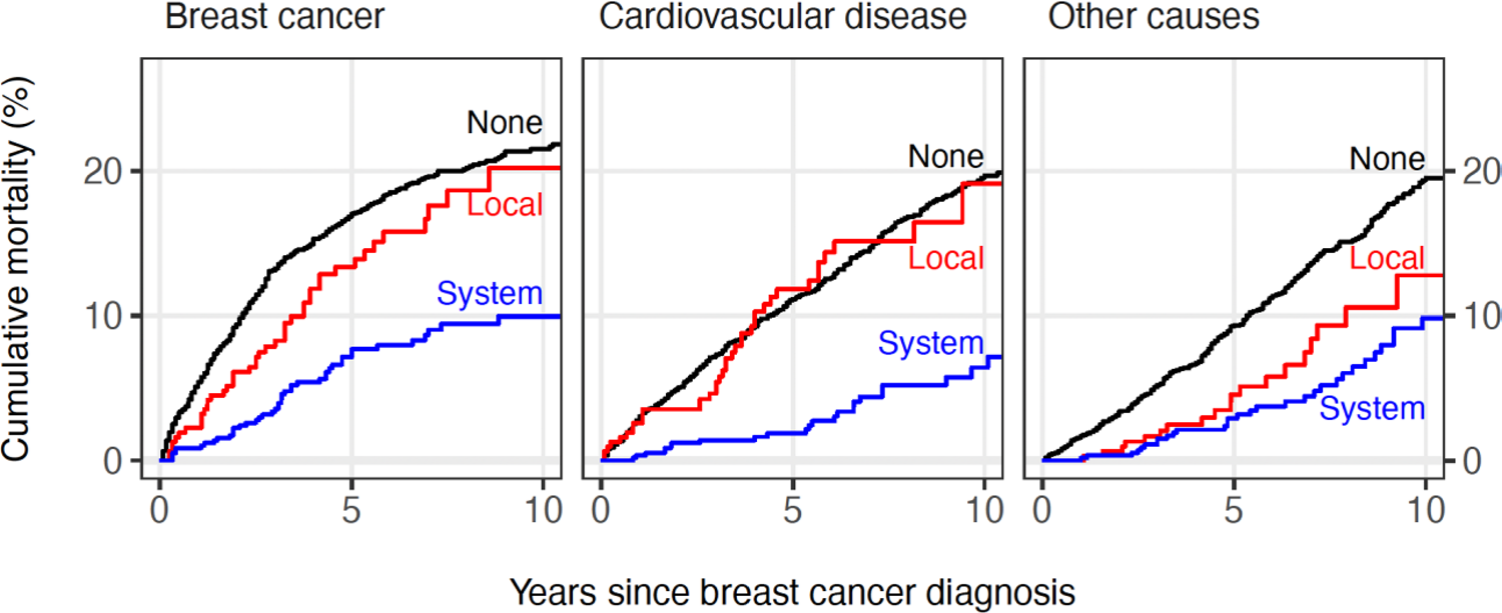
The cumulative mortality curves to breast cancer, cardiovascular diseases, and other causes among systemic, local, and non-users of the menopausal hormone therapy.

The unadjusted 10-year cumulative mortality due to breast cancer was 10% among systemic MHT users and 20% among local MHT users, and 22% among non-MHT users (Fig. 2). The 10-year mortality due to cardiovascular disease (CVD) was 6% among systemic MHT users, 19% among local MHT users, and the mortality due to other causes was 10% among systemic MHT users and 13% among local MHT users, and 19% among non-MHT users.

In the Cox proportional hazards model, the mortality due to breast cancer appeared to be lower among systemic MHT users (hazard ratio [HR]: 0.49, 95% confidence interval [Cl] 0.36–0.67) compared with non-MHT users (Table 2, Supplementary Fig. 1). However, among local MHT users, the result was inconclusive (HR: 0.93, 95% CI 0.68–1.27). The mortality due to both CVD and other causes was found to be reduced among systemic MHT users compared to non-MHT users, respectively (HR 0.49, 95% Cl 0.32–0.74, and HR 0.51, 95% CI 0.35–0.76). The mortality due to CVD was not observed to be different among local MHT users compared with non-MHT users (HR 1.0595% CI 0.75–1.48); the mortality due to other causes was shown to be reduced (HR 0.56 95% CI 0.350.9; Table 2).

**Table 2:** Estimation results from Cox proportional hazard models of mortality from breast cancer, cardiovascular diseases, and other causes.

	Breast cancer		Cardiovascular diseases		Other causes	
	Cases	HR (95% CI)	Cases	HR (95% Cl)	Cases	HR (95% CI)
Year of BC diagnosis						
2000–2003	160	1 (reference)	161	1	167	1
2004–2007	174	0.97 (0.78–1.21)	151	1.08 (0.85–1.37)	121	0.81 (0.64–1.04)
2008–2011	178	0.59 (0.79–1.24)	95	0.87 (0.65–1.15)	67	0.68 (0.50–0.93)
Age at BC diagnosis (years)						
40–59	57	1.15 (0.79–1.69)	10	0.38 (0.18–0.78)	24	0.88 (0.51–1.53)
60–64	56	1.20 (0.82–1.76)	19	0.79 (0.44–1.41)	25	1.05 (0.61–1.81)
65–69	50	1	27	1	27	Ref
70–74	74	1.40 (1.19–2.43)	49	2.26 (1.41–3.62)	52	2.37 (1.49–3.78)
75–79	109	2.35 (1.68–3.28)	91	4.21 (2.73–6.48)	79	3.63 (2.34–5.62)
80–84	89	2.81 (1.98–3.97)	109	8.18 (5.35–12.52)	75	5.72 (3.67–8.89)
85–100	77	3.90 (2.73–5.58)	102	14.27 (9.26–21.99)	73	11.16 (7.13–17.47)
90–100	24	5.89 (3.59–9.68)	25	14.50 (8.26–25.45)	23	15.41 (8.69–27.33)
Duration of T2D (years)						
0.5–< 3	119	1	54	1	70	1
3–< 6	101	0.95 (0.73–1.24)	69	1.42 (1.00–2.03)	64	1.03 (0.73–1.44)
6–< 12	167	1.05 (0.83–1.33)	144	2.00 (1.46–2.74)	126	1.32 (0.99–1.77)
12–< 42	125	1.33 (1.03–1.71)	140	3.56 (2.60–4.87)	95	1.92 (1.41–2.61)
Stage						
Local	93	1	196	1	194	1
Advanced	394	5.79 (4.61–7.25)	162	1.21 (0.98–1.49)	128	1.00 (0.80–1.25)
Unknown	25	2.42 (1.56–3.77)	49	2.46 (1.80–3.37)	33	1.72 (1.19–2.49)
Prediagnostic MHT use						
None	422	1	343	1	306	1
Systemic	45	(0.26–0.48)	25	0.22 (0.15–0.33)	30	0.29 (0.20–0.42)
Local	45	0.78 (0.58–1.07)	39	0.90 (0.64–1.25)	19	0.50 (0.31–0.79)

*BC* Breast Cancer, *CI* Confidence Interval, *HR* Hazard Ratio, *T2D* Type 2 diabetes, *MHT* menopausal hormone therapy.

## Discussion

We found that, among breast cancer patients with T2D, prediagnostic use of systemic MHT predicted lower mortality due to breast cancer, cardiovascular diseases and other causes of death compared to non-MHT users. However, the mortality due to breast cancer or cardiovascular diseases were not found to be different among women who used only local MHT or who did not use MHT at all. This is in line with the previous data that the systemic MHT use decreases the rate of hypercholesterolemia and triglyceridemia in postmenopausal women^25^. Again, there is no evidence that local MHT would be able to have clinically significant impact on serum estrogen levels and consequently any cardioprotective effects. As far as we know, there are no previous reports on the association between the prediagnostic use of MHT and breast cancer survival in women with T2D.

The main strength of our study is the use of nationwide database registers that contain precise information about the timing of breast cancer diagnoses. The quality of data in Finnish registers is high, including those of the Finnish Hospital Discharge Register^26^. The Finnish nationwide diabetes (FinDM) database contains information about the date and quantity of all medication prescribed by doctors and reimbursed by Social Insurance Institution, including antidiabetic medication and MHT, starting from 1994. All the Nordic cancer registries have high-quality standards for the comprehensiveness and accuracy of the registered data, and patients’ causes of death are collected from the national cause of death registries in all Nordic cancer registries^27^. Moreover, practices and procedures of the Cause of Death Register of Finland comply with the coding of causes of death for mortality statistics^28^. The Finnish Cancer Registry (FCR) data made it possible to identify cancer-specific and other causes of death^27^. The duration of T2D is also precise because it is based on the first recorded T2D diagnosis in any of the incorporated registers or the first purchase of any form of antidiabetic medication (ADM). Moreover, we relied on medical records instead of interviewing patients regarding their MHT use, which is an important difference between most previous studies and ours.

We have some limitations in our study. The data was available only from the registers, which lacked information on traditional breast cancer prognostic factors, including hormone receptor status and tumor size. It has been suggested that hormone receptor status and grade are variables that reflect a biological effect of MHT on tumors, whereas tumor size and lymph node status represent time-dependent variables, which are more sensitive to detection bias^29^. The FCR lacks specific data on breast cancer treatment. Also, data on socioeconomic status was not available in our study. Furthermore, some local MHT can be purchased without a prescription, therefore not recorded into the Prescription Register, which may have affected our results in terms of local MHT use. We hypothesize that we may have observed an even greater reduction in mortality among prediagnostic local MHT users because of the healthy user bias. In our study, MHT use was studied over a 5-year period before breast cancer diagnosis and, therefore, women who used MHT and discontinued treatment more than five years before breast cancer diagnosis are classified into the “no MHT history’ group. Again, at least 180-day use of the MHT was required to be included in the MHT user group. Finally, the women using systemic MHT were older than the non-users and the users of local MHT, as expected. Therefore, systemic MHT users may seem healthier than they are, despite Cox proportional hazard models being adjusted with age at breast cancer diagnosis.The association between cancer-specific mortality and MHT use among breast cancer patients has been assessed in many cohort studies^11–18,30^. Yu et al. have shown in a meta-analysis that prediagnostic MHT use was associated with decreased risk of dying from breast cancer (HR 0.88, 95% CI 0.81–0.97) or any cause (HR 0.79, 95% CI 0.69–0.90)^31^. On the other hand, some cohort studies have reported no clear evidence of the association between MHT use and cancer-specific mortality among breast cancer patients^18,30^. Several cohort studies observed the reduction of breast cancer or all-cause mortality only among current MHT use but not among past MHT users^12,13,17,18,30^. No previous studies have focused on women with both T2D and breast cancer. Furthermore, many previous studies did not classify MHT use as systemic and local; however, some categorized MHT use into estrogen alone or in combination with progestin. Our data consisted of 13 different MHT compounds and thus the effect of each compound was not able to be specified.

The cumulative exposure of breast tissue to estrogen is a major risk factor for breast carcinogenesis, and the most known breast cancer risk factors, including younger age at menarche, older age at menopause, hormonal birth control medications, and prolonged systemic MHT, are linked to estrogen exposure^32^. In line with this, in high-risk genetic variant carriers, the most effective intervention to decrease breast cancer incidence and mortality is salpingo-oophorectomy, in addition to risk-reducing mastectomy^33,34^.

Healthy user bias is present in all observational studies concerning MHT. MHT users tend to be healthier than non-MHT users, making MHT appear more beneficially effective. It is well recognized that women at higher educational and socioeconomic levels are more likely to be diagnosed with breast cancer^35^. Further, an early stage at breast cancer diagnosis among MHT users has been proposed to be the reason for favorable breast cancer survival for MHT users^36^. This may at least partly be explained by educational and/or socioeconomic level, a higher probability of screening, and younger age among MHT users compared to non-MHT users rather than a biological effect of MHT^37^. A cohort study reported that women with breast cancer who previously used MHT less than 5 years prior did not show clear increased breast cancer mortality; contrastingly, past users with longer prior MHT use showed increased breast cancer mortality over the next 20 years^38^.

The standard recommendation for the duration of MHT use has been 5 years or less and the treatment initiation is not recommended for individuals older than age 60^39^. A meta-analysis of randomized controlled trials examining the timing hypothesis for the MHT initiation and CVD risk supported the importance of the timing of initiation of MHT. It was concluded that MHT might have favorable effects on mortality due to all causes and CVD only in post-menopausal women under 60 years of age^40^. However, disagreements remain considering the CVD-related risks and benefits of using MHT. Thus, further work needs to be done considering the dose, route of MHT, timing after menopause, duration of MHT use, other hormone effects, and age.

In conclusion, there was reduced breast cancer mortality among prediagnostic systemic MHT users compared with non-MHT users in women with breast cancer and T2D. The cardiovascular and other causes of mortality were also decreased among prediagnostic systemic MHT users. Our results suggest that prediagnostic use of MHT in T2D patients does not have an unfavorable effect on breast cancer prognosis.

## Methods

The Strengthening the Reporting of Observational Studies in Epidemiology (STROBE) guidelines were followed in the current study^41^. The data on women with diabetes were assembled from the FinDM database. The FinDM database combines data from multiple nationwide registers, including the Special Refund Entitlement Register and the Prescription Register from the Social Insurance Institution, the Care Register for Health Care from the Finnish Institute for Health and Welfare, and the Causes of Death Register from Statistics Finland^42^.

A patient is entered in the FinDM database either at the time of diabetes diagnosis or at the time of the first reimbursement for antidiabetic medication in some of the registers^42^. Data on in-hospital records of diagnosis are available from 1969 for inpatients and 1998 for outpatients^42^. The categorization of patients into type 1 and type 2 diabetes is predominantly based on the ADM used as the first-line treatment. In comparison to a local diabetes register, the FinDM data possess good coverage of diabetes patients^43^. The FinDM database records are linked with information from FCR. This has made cancer data, such as information on cancer stages, readily available since 1953^27^. The cancer stage at diagnosis is grouped in the FCR as 0) unknown, (1) localized, (2) non-localized, only regional lymph node metastases, (3) metastasized or invades adjacent tissues, (4) non-localized, no information on extent, (5) locally advanced, tumor invades adjacent tissues, and (6) non-localized, also distant lymph node metastases. In our study, the stage coding was 0) unknown, 1) local, 2–6) advanced.

From the FinDM database, we first identified 13,804 women with T2D who had also been diagnosed with breast cancer. Inclusion criteria for our study cohort were women (1) who were at least 40 years old when diagnosed with T2D, (2) whose breast cancer was diagnosed between 1 January 2000 and 31 December 2011, (3) who had no previous cancers, (4) in whom the estimated duration of T2D was at least 180 days before breast cancer diagnosis, and (5) whose breast cancers were diagnosed before autopsy. The final study cohort contained 3189 women with T2D and breast cancer (Fig. 1).

The patients were classified into the following mutually exclusive groups, according to their use of MHT within the 5-year period before breast cancer diagnosis: (1) systemic MHT; (2) local MHT; (3) no MHT history (Supplementary file). Systemic MHT is contraindicated after breast cancer diagnosis in Finland. Systemic MHT covered oral drugs, transdermal patches, gels and implants. Local MHT included vaginal creams, vaginal tablets, pessaries and rings. A patient was categorized as a systemic MHT user after purchasing systemic MHT for a time period of > 180 days regardless of the use of local hormonal treatment. A patient was categorized as a local MHT user after purchasing local hormonal treatment for the time period of > 180 days if there was no history of systemic MHT purchase. If a patient had purchased systemic MHT or local hormonal treatment for a time period of less than 180 days, she was classified as the “no MHT history “group. Patients who did not purchase either of them were also classified into this group. Follow-up of the study cohort began on the date of breast cancer diagnosis and concluded on the date of death, emigration or closure of the follow-up period (31 December 2013), whichever occurred first. We used the FCR data to gather the follow-up information. FCR records are annually matched with the Cause of Death Register maintained by Statistics Finland via computerized links based on personal identity codes to add the dates and causes of death to the FCR records. The assessment of each cancer patient’s cause of death is based on all data available in the FCR record, and on that basis, the FCR personnel judges whether the patient died due to cancer or from some other cause. In this study, the causes of death were classified into two groups: death due to breast cancer and death due to other causes. Deaths due to other causes were then divided into two subgroups: death due to cardiovascular diseases (International Classification of Diseases 10th Revision [ICD-10] codes I00-I99) and death due to other causes. Additionally, FCR records are regularly linked to data in the Population Register Centre of Finland, which obtains information on individuals’ emigration and official place of residence prior to the date of diagnosis^27^.

We used the Aalen-Johansen estimator of cumulative incidence function for competing risks in the different medication groups for a graphical description of the cumulative mortality due to the three causes of death separately^44,45^. In order to adjust for the effects of calendar year, age and stage at the time of breast cancer diagnosis, and T2D duration, the Cox proportional hazard models were fitted for the three causes of death individually, and HRs (with accompanying 95% Cls) in the medication groups were evaluated via the adjusted Cox models. For model diagnostics, both cloglog plots and plots of the scaled Schoenfeld residuals were visually reviewed^46^. Notably, no evidence of a violation of the proportional hazard’s assumption could be detected as having had any essential impact on the inference. R environment (version 4.1.0) was used throughout for statistical analyses^47^. The Cox models were adjusted, and the assumptions were scrutinized using the survival package functions^48^.

All procedures performed in this study involving human participants were in accordance with the ethical standards of the Finnish National Research Committee and the 1964 Declaration of Helsinki and its later amendments. According to Finnish legislation, no separate ethics approval is needed for studies involving only administrative registers. However, ethics approval was obtained for this FinDM study from the research ethics committee of the Finnish Institute of Health and Welfare (30 January 2014, meeting 1/2014, 340 §609). Permission to use data was obtained from those maintaining the original registers (i.e. Finnish Institute for Health and Welfare, the Social Insurance Institution, and Statistics Finland). Need for informed consent was waived by research ethics committee of the Finnish Institute of Health and Welfare.

### Supplementary Information

Supplementary Figure 1.

Supplementary Information 2.

## Data Availability

The datasets analyzed during the current study are not publicly available due to confidentiality reasons, but anonymized data collected into tables are available from the corresponding author on reasonable request.

## References

[1] Shao S (2018). Diabetes and overall survival among breast cancer patients in the US military health system. Cancer Epidemiol. Biomarkers Prev..

[2] Lee KN (2020). Type 2 diabetes, breast cancer specific and overall mortality: Associations by metformin use and modification by race, body mass, and estrogen receptor status. PLoS ONE.

[3] Luo J (2015). Pre-existing diabetes and breast cancer prognosis among elderly women. Br. J. Cancer.

[4] Nelson HD (2004). Commonly used types of postmenopausal estrogen for treatment of hot flashes: scientific review. JAMA.

[5] Maclennan AH, Broadbent JL, Lester S, Moore V (2004). Oral oestrogen and combined oestrogen/progestogen therapy versus placebo for hot flushes. Cochrane Database Syst. Rev..

[6] The 2022 Hormone Therapy Position Statement of The North American Menopause Society” Advisory Panel. The 2022 hormone therapy position statement of The North American Menopause Society. *Menopause* **29**, 767–794 (2022). 10.1097/GME.0000000000002028 35797481

[7] Stuenkel CA, Davis SR, Gompel A, Lumsden MA, Murad MH, Pinkerton JV, Santen RJ (2015). Treatment of symptoms of the menopause: An endocrine society clinical practice guideline. J. Clin. Endocrinol. Metab..

[8] McVicker L, Labeit AM, Coupland CAC (2024). Vaginal estrogen therapy use and survival in females with breast cancer. JAMA Oncol..

[9] Beral V, Million (2003). Women Study Collaborators. Breast cancer and hormone-replacement therapy in the Million Women Study. The Lancet.

[10] Henderson BE, Paganini-Hill A, Ross RK (1991). Decreased mortality in users of estrogen replacement therapy. Arch. Intern. Med..

[11] Fletcher AS (2005). Use of hormone replacement therapy (HRT) and survival following breast cancer diagnosis. Breast.

[12] Newcomb PA (2008). Prediagnostic use of hormone therapy and mortality after breast cancer. Cancer Epidemiol. Biomarkers Prev..

[13] Rosenberg LU (2008). Menopausal hormone therapy in relation to breast cancer characteristics and prognosis: A cohort study. Breast Cancer Res..

[14] Rauh C (2015). Hormone therapy and its effect on the prognosis in breast cancer patients. Geburtshilfe Frauenheilkd.

[15] Schuetz F (2007). Reduced incidence of distant metastases and lower mortality in 1072 patients with breast cancer with a history of hormone replacement therapy. Am. J. Obstet. Gynecol..

[16] Sener SF (2009). The effects of hormone replacement therapy on postmenopausal breast cancer biology and survival. Am. J. Surg..

[17] Obi N (2016). Relationship between menopausal hormone therapy and mortality after breast cancer The MARIEplus study, a prospective case cohort. Int. J. Cancer.

[18] Strickland DM, Gambrell RD, Butzin CA, Strickland K (1992). The relationship between breast cancer survival and prior postmenopausal estrogen use. Obstet. Gynecol..

[19] Manson JE (2013). Menopausal hormone therapy and health outcomes during the intervention and extended poststopping phases of the Women’s Health Initiative randomized trials. JAMA.

[20] Anagnostis P (2019). Menopausal hormone therapy and cardiovascular risk: Where are we now?. Curr. Vasc. Pharmacol..

[21] Wolf PH, Madans JH, Finucane FF, Higgins M, Kleinman JC (1991). Reduction of cardiovascular disease-related mortality among postmenopausal women who use hormones: Evidence from a national cohort. Am. J. Obstet. Gynecol..

[22] Grodstein F (2000). A prospective, observational study of postmenopausal hormone therapy and primary prevention of cardiovascular disease. Ann. Intern. Med..

[23] Petitti DB, Sidney S, Quesenberry CP (2000). Hormone replacement therapy and the risk of myocardial infarction in women with coronary risk factors. Epidemiology.

[24] Rossouw JE (2002). Risks and benefits of estrogen plus progestin in healthy postmenopausal women: Principal results from the women’s health initiative randomized controlled trial. JAMA.

[25] Nie G (2022). The effects of menopause hormone therapy on lipid profile in postmenopausal women: A systematic review and meta-analysis. Front. Pharmacol..

[26] Sund R (2012). Quality of the finnish hospital discharge register: A systematic review. Scand. J. Public Health.

[27] Pukkala E (2018). Nordic cancer registries—An overview of their procedures and data comparability. Acta Oncol..

[28] Lahti RA, Penttilä A (2001). The validity of death certificates: Routine validation of death certification and its effects on mortality statistics. Forensic Sci. Int..

[29] Stahlberg C (2005). Breast cancer incidence, case-fatality and breast cancer mortality in Danish women using hormone replacement therapy–a prospective observational study. Int. J. Epidemiol..

[30] Ewertz M, Gillanders S, Meyer L, Zedeler K (1991). Survival of breast cancer patients in relation to factors which affect the risk of developing breast cancer. Int. J. Cancer.

[31] Yu X (2017). Hormone replacement therapy and breast cancer survival: A systematic review and meta-analysis of observational studies. Breast Cancer.

[32] Britt KL, Cuzick J, Phillips KA (2020). Key steps for effective breast cancer prevention. Nat. Rev. Cancer.

[33] Zaluzec EK, Sempere LF (2024). Systemic and local strategies for primary prevention of breast cancer. Cancers.

[34] Kotsopoulos J, Gronwald J, Huzarski T (2024). Bilateral oophorectomy and all-cause mortality in women with *BRCA1* and *BRCA2* sequence variations. JAMA Oncol..

[35] Palme M, Simeonova E (2015). Does women’s education affect breast cancer risk and survival? Evidence from a population based social experiment in education. J. Health Econ..

[36] Magnusson C, Holmberg L, Nordén T, Lindgren A, Persson I (1996). Prognostic characteristics in breast cancers after hormone replacement therapy. Breast Cancer Res. Treat.

[37] Godina C (2020). Prognostic impact of menopausal hormone therapy in breast cancer differs according to tumor characteristics and treatment. Front. Oncol..

[38] Green J (2019). Cohort profile: The million women study. Int. J. Epidemiol..

[39] Harman SM (2011). Timing and duration of menopausal hormone treatment may affect cardiovascular outcomes. Am. J. Med..

[40] Nudy M, Chinchilli VM, Foy AJ (2019). A systematic review and meta-regression analysis to examine the ‘timing hypothesis’ of hormone replacement therapy on mortality, coronary heart disease, and stroke. Int. J. Cardiol. Heart Vasc..

[41] von Elm E (2008). The strengthening the reporting of observational studies in epidemiology (STROBE) statement: Guidelines for reporting observational studies. J. Clin. Epidemiol..

[42] Arffman, M. *et al.* FinDM database on diabetes in Finland. (2020).

[43] Sund R, Harno K, Ranta S, Tolppanen E (2010). Evaluation of case inclusion in two population-based diabetes registers. Finn. J. eHealth eWelfare.

[44] Putter H, Fiocco M, Geskus RB (2007). Tutorial in biostatistics: Competing risks and multi-state models. Stat. Med..

[45] deGlas NA (2016). Performing survival analyses in the presence of competing risks: A clinical example in older breast cancer patients. J. Nat. Cancer Inst..

[46] Grambsch PM, Therneau TM (1994). Proportional hazards tests and diagnostics based on weighted residuals. Biometrika.

[47] Therneau, T. _A Package for Survival Analysis in S_version 2.38. (2015).

[48] Team, R. C. *R: A language and environment for statistical computing*. (R Foundation for Statistical Computing, 2017).

