# Supplementary material for: The association of breast cancer patients survival and prior menopausal hormone therapy in women with type 2 diabetes
Source: Sci Rep. 2024 Jul 16;14:16478. doi: 10.1038/s41598-024-65916-2 (PMC11252321; doi:10.1038/s41598-024-65916-2)
Supplement: Supplementary file 1 — Supplementary Figure 1. [file 41598_2024_65916_MOESM1_ESM.pdf]

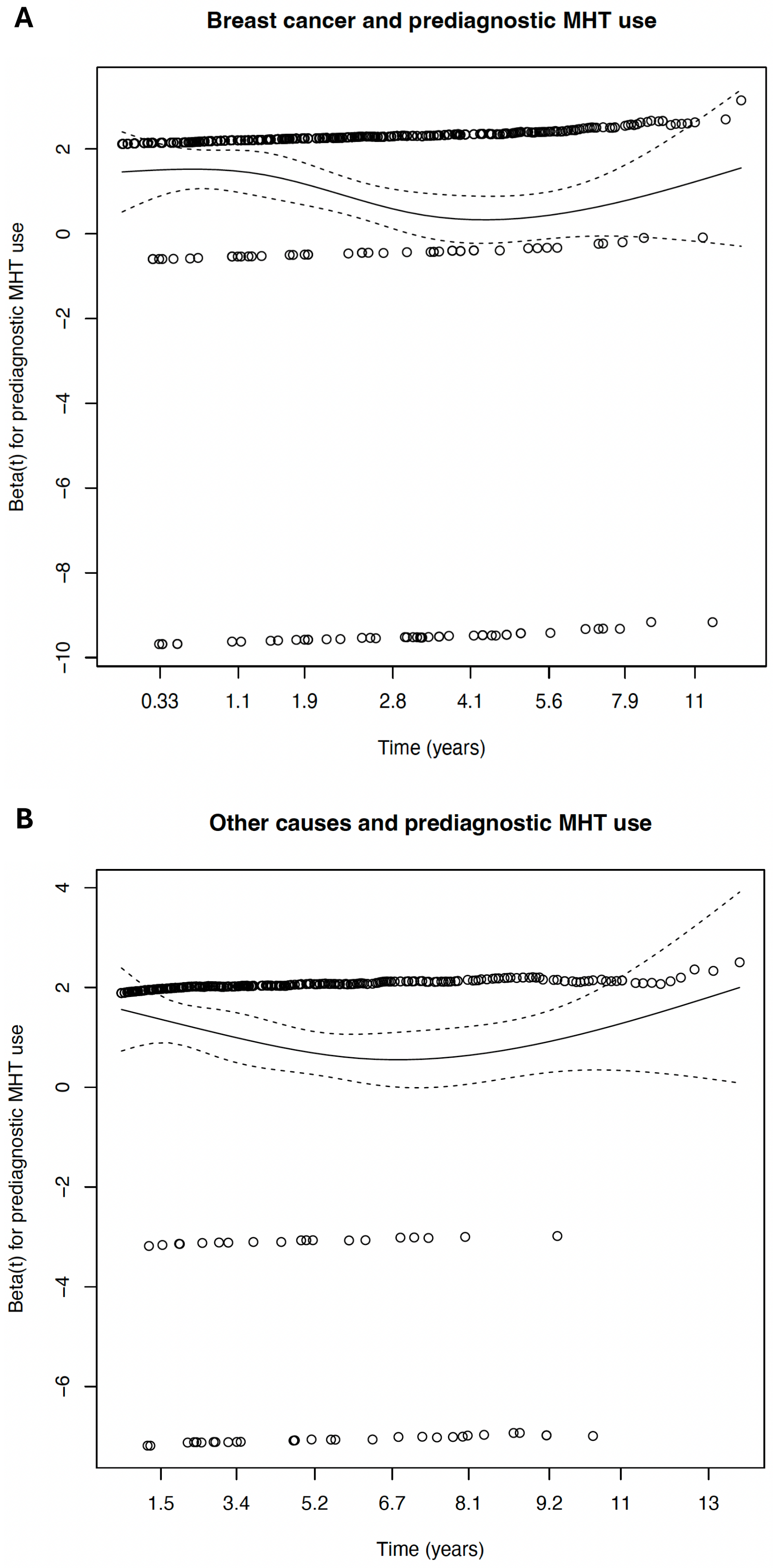

Supplementary Figure 1. Schoenfeld residual plots from breast cancer-specific mortality (A) and the mortality from other causes (B).
