# Supplementary material for: The association of breast cancer patients survival and prior menopausal hormone therapy in women with type 2 diabetes
Source: Sci Rep. 2024 Jul 16;14:16478. doi: 10.1038/s41598-024-65916-2 (PMC11252321; doi:10.1038/s41598-024-65916-2)
Supplement: Supplementary file 2 — Supplementary Information 2. [file 41598_2024_65916_MOESM2_ESM.pdf]

**Supplementary File**: ATC codes for different types of menopausal hormone therapy (MHT)

**Menopausal hormone therapy**
**Systemic**

| | |
|---|---|
| G03FA01 | oestradiol/norethisterone |
| G03FA17 | drospirenone/oestradiol |
| G03CA03 | oestradiol |
| G03FB06 | oestradiol/medroxyprogesterone |
| G03FB05 | oestradiol/norethisterone |
| G03FA16 | oestradiol/norethisterone |
| G03FB08 | oestradiol/dydrogesterone |
| G03FA14 | oestradiol/dydrogesteron |
| G03FA12 | oestradiol/medroxyprogesterone |
| G03FB01 | oestradiol/norethisterone |
| G03CX01 | tibolone |
| G03FA01 | dydrogesterone |
| G03CA57 | oestroradiol |

**Local**

| | |
|---|---|
| G03CA03 | oestradiol |
| G03CA04 | oestradiol |
